# Stronger color evasive racial ideologies predict lower likelihood of open adoption placement with same-sex couples

**DOI:** 10.3389/fsoc.2024.1270527

**Published:** 2024-07-25

**Authors:** Brian J. Reece, Diana L. Jenkins, Austin C. Folger, Daniel S. Shaw, Jenae M. Neiderhiser, Jody M. Ganiban, Leslie D. Leve

**Affiliations:** ^1^Department of Counseling Psychology & Human Services, University of Oregon, Eugene, OR, United States; ^2^Prevention Science Institute, University of Oregon, Eugene, OR, United States; ^3^Department of Psychology, University of Pittsburgh, Pittsburgh, PA, United States; ^4^Department of Psychology, The Pennsylvania State University, State College, University Park, PA, United States; ^5^Department of Psychological and Brain Sciences, The George Washington University, Washington, DC, United States

**Keywords:** racism, homophobia, social justice, adoption, parent

## Abstract

Although the adoption rate among same-sex couples has been increasing, limited research has focused on factors influencing decision making related to placing children with such couples, particularly from the standpoint of birth mothers. Additionally, there is a gap in the literature regarding how biases may influence birth mothers’ decision to place their child with a same-sex couple. This study sought to examine the association between birth mothers’ racial ideologies and their decision to voluntarily place their children with same-sex couples (*n* = 29) or mother–father couples (*n* = 354) during the adoption process. Results indicated that birth mothers with stronger color evasive racial attitudes were significantly less likely to place their children with same-sex couples. The need for additional research about the intersections among various forms of bias in the adoption process and the effect of potential interactions between homophobia and racism are discussed. Suggestions for professionals wishing to minimize homophobic and racist bias are provided.

## 1 Introduction

Over time, there has been a shift in the United States regarding the acceptability of same-sex marriage, with individuals becoming more supportive (Baunach, 2012; Lian, 2022). This may reflect a promising decrease in explicit negative views toward marginalized sexual identities. However, according to a study examining views among straight individuals regarding lesbian women and gay men, decreases in negative implicit biases appear to be decreasing more slowly than decreases in negative explicit views (Westgate et al., 2015). Staats et al. (2017) argued that implicit biases are involuntary, vigorous, and stalwart, and that most Americans of all races tend to exhibit unconscious racist attitudes. It is important to examine these biases because they are typically associated with discriminatory behaviors (Cameron et al., 2012; Greenwald et al., 2022).

Implicit associations are difficult to recognize, suggesting that one cannot simply eliminate a particular bias (Dasgupta, 2013). Research by Sabin et al. (2015) on explicit and implicit biases toward lesbians and gay men among straight health care providers exemplifies this issue. Specifically, even though individuals expressed less explicit homophobic views, their unconscious biases always favored straight individuals over lesbian and gay individuals. However, investigations regarding implicit biases that can negatively affect sexual minorities have focused predominantly on health care settings. Little attention has been given to how implicit biases can affect other areas of one’s life, such as decisions related to the selection of families in adoption processes.

Even though access to adoptions for same-sex couples increased following a 2015 Supreme Court ruling which effectively legalized same-sex adoption across all of the United States, most research on parenting and adoption has focused on straight couples. A growing body of evidence demonstrates that gay and lesbian parents show few differences from hegemonic two-parent (i.e., mother–father) structures on a number of parent/child mental and physical health outcomes (Goldberg and Smith, 2013; Manning et al., 2014; Bos et al., 2016; Reczek et al., 2016; Mazrekaj et al., 2022). Thus, the myth of the “dangerous gay parent” that has been rampant in popular culture appears to be unfounded, highlighting the importance of examining relevant constructs and research questions in the area of parenting and bias (Averett et al., 2009; Patterson and Farr, 2022).

Prior studies have highlighted that queer couples experience a variety of barriers when engaging in the adoption process. Specifically, Goldberg et al. (2019) found that the bias that sexual minorities experienced from adoption and legal agencies as well as birth families interfered with the timing and success of an adoption. Additionally, sexual minority adoptive parents have stated that bias from both the birth parent and the adoption agency hindered the adoption process (Ryan and Whitlock, 2008; Goldberg et al., 2012; George, 2016). Brodzinsky et al. (2002), for example, found that agency religious affiliation, public vs. private status, and even the gender of the staff created real barriers for same-sex couples even being considered as adoptive parents. The barriers are even greater for sexual minority adoptive parents of color and those with fewer financial resources (Goldberg, 2023). One explanation for this bias by birth parents could be the notion that individuals place their children with those that are “like them.” In other words, heterosexual birth mothers may place children with families that are also heterosexual and may exhibit bias toward same-sex couples.

Still, studies of queer adoptive couples focus predominantly on the *experience* or *effect* of bias from the standpoint of marginalized sexualities. The research questions focus on experiences and outcomes of gay adoptive parents and their adopted children, often comparing them to those of straight adoptive parents and their adopted children (e.g., Goldberg et al., 2011a; Farr and Vázquez, 2020b; Mazrekaj et al., 2022). While this research is important, it does not elucidate what causes these outcomes nor how discriminatory practices can be mitigated or stopped altogether. By focusing solely on the experience of bias (i.e., the “what”), we can only partially advance our understanding of the structure and nature of bias itself (i.e., the “how” and the “why”) and are missing insight into how to create a more inclusive and just adoption practice.

Social scientists have increasingly examined the complex relationships between and among a variety of identities (Seng et al., 2012). The current landscape of adoption-related literature across disciplines includes a growing body of research on such topics, including race (e.g., Smith et al., 2011; Ung et al., 2012; White et al., 2022), sexuality (e.g., Goldberg et al., 2011b; Kranz, 2020; Goldberg, 2023), and emerging knowledge on the intersection of social identities (e.g., Dorow and Swiffen, 2009; Johnson et al., 2020). Less research, however, has been conducted to examine the relationships among different types of bias (Neville et al., 2000).

One form of bias that may be associated with other forms of bias is color evasive racism, which refers to the general avoidance of race (Frankenberg, 1993; Neville et al., 2013). Color evasion occurs when individuals do not acknowledge race or state that they do not “see color.” At an individual level, this approach serves either to (1) not appear racist or biased and/or (2) minimize racial bias without cost to oneself or one’s group (Neville et al., 2013). This strategic, and almost benevolent-appearing, approach to race may be used to avoid conversations of racism and thus the acknowledgement of one’s power and privilege, but it has harmful consequences. For instance, color evasion functions as a form of microaggression and does not effectively reduce the appearance of being biased (Babbitt et al., 2016; Mekawi and Todd, 2018). One study found that white employees’ color evasion endorsement was associated with lower work engagement with racial and ethnic minority employees (Plaut et al., 2009), suggesting that color evasion can still appear biased and harmful to those from racial and ethnic minority groups.

The emergence of critical research and scholarship over the last several decades, influenced by a variety of theories (e.g., critical race, queer, feminist, and postcolonial theories), has provided frameworks for the social sciences to reflect the complex and diverse range of human thought and experience in society. Our understanding of the experience and effects of bias and the complex nature of intersecting identities is a direct result of the diversifying perspectives of researchers in the social sciences (e.g., Kurdek, 2005; Braveman et al., 2011; Slopen et al., 2012; Evans and Kim, 2013) in addition to the work of scholars in other disciplines (e.g., Butler, 2006; Denetdale, 2006; Deer, 2010; Kusalik, 2010). Alongside theorists who described the influence of standpoint and epistemology on our understanding the experience of bias (Collins, 1990; Harding, 1998), others have implored scholars and activists to adopt an intersectional approach, one that does not compartmentalize the interlocking identities we possess, but rather embraces their inseparability (e.g., Crenshaw, 1991).

Beyond the experience of bias (i.e., the “what”), research that focuses on the structure and nature of bias is needed (i.e., the “how” and the “why”). For example, there is evidence that women are less biased than men against gays and lesbians (Whitley and Kite, 1995) and that this relationship is moderated by race (Vincent et al., 2009). Moving beyond our understanding of simply the experience of bias, we can begin to understand how bias manifests structurally by the ways in which it interacts with other parts of the system. In this case, the evidence suggests that our society socializes women in ways differently than men—that is, to be more accepting of non-dominant sexualities (Whitley and Kite, 1995; Butler, 2006). While women may be less likely to be biased against gays and lesbians than men, other factors may also be at play. For example, Averett et al. (2011) found that a variety of factors predicted more bias against gay and lesbian adoptive parents, including higher levels of religiosity and political ideology. Results also differed when comparing married mothers to unmarried mothers, with association with Christianity being predictive for married mothers while not being predictive for unmarried mothers.

The picture is even more complex when we concurrently consider race and/or any other identity status. Just as women are less likely than men to be biased against gays and lesbians, they are also less likely than men to exhibit color evasive racial ideologies, defined here as a “dominant racial ideology or worldview that serves to justify and explain away racial inequalities in society,” (Neville et al., 2013, p. 458). Although this does not imply that women are necessarily less racially biased or are less likely to exhibit feelings of racial superiority than men, evidence suggests that color-evasive racial ideologies may indeed be significantly correlated with both racial and gender-based bias (Neville et al., 2000). In other words, the various forms of bias against one group or another are not mutually exclusive, especially considering that bias against one group (e.g., women) may indeed be related to bias against another (e.g., lesbians) by the very nature of the group identities overlapping to some extent.

Color evasive racial ideologies (Annamma et al., 2017) have been associated with a range of other constructs, including social dominance orientation (Worthington et al., 2008; Daughtry et al., 2020), lower cultural appreciation (Spanierman et al., 2008), lower cultural empathy (Burkard and Knox, 2004; Yi et al., 2023), and victim-blaming ideology (Marshall, 2012; Wang et al., 2023). Furthermore, a study of the relation between genetic lay theories regarding race and sexual orientation and their impact on bias and discrimination against these groups found fairly strong correlations between a variety of forms of anti-Black bias and discrimination and anti-gay/anti-lesbian bias and discrimination (Jayaratne et al., 2006).

Thus, color evasion is a form of bias that could intersect other forms of bias, such as homophobic attitudes. Yet, no research has focused on how color evasion is associated with bias toward sexual minorities. Such research could demonstrate the compounding effects of multiple biases and their influence on discriminatory behaviors.

To our knowledge, one context lacking research is the exploration of the decision-making process that birth mothers use when choosing whether to place their children with same-sex adoptive parents. This study sought to examine such decisions and whether they have an association with color evasion. Specifically, this study measured color evasion (through the Color-Blind Racial Attitudes Scale) and its interaction with the selection of same-sex or mother–father couples within the adoption process. An important aspect of this study is the inclusion of same-sex couples in the pool of adoptive parents and the generally open nature of the selection process that birth parents engage in when choosing an adoptive family.

With implicit bias as a guiding framework (Staats et al., 2017), this study, which was exploratory in nature given the lack of current research on the topic, examined the relationship between the selection of same-sex or mother–father adoptive family configurations and birth mothers’ levels of color evasion (Neville et al., 2000). It was hypothesized that stronger color evasion would be associated with the choice of mother–father parent adoptive families more frequently than same-sex parent adoptive families.

## 2 Methods

### 2.1 Procedures and participants

This study used existing data from the Early Growth and Development Study (EGDS; Leve et al., 2019). EGDS is a prospective study of adoptive and birth families that seeks to understand the peer, family, and social contexts and influences that affect child development, in addition to genetic factors inherited from birth parents. EGDS drew participants from adoption agencies that served a range of open and closed adoptions, although nearly all adoptions were at least partially open, meaning that birth and adoptive families shared some information about each other. Agencies often served as the facilitator of contact, by forwarding letters and pictures between families; other birth and adoptive families had direct contact with each other before and after the placement. Matches between birth parents and adoptive families typically occurred with the help of the agency. Most often, birth parents selected the adoptive family based on profiles the adoptive parents created and by meeting them in person. In instances where birth parents did not wish to select the family, agencies followed their own policy for family selection.

The EGDS full sample included 561 linked sets of adoptive and birth families. We used data from 383 birth mother-adoptive parent dyads whereby the birth mother had completed a measure of CBRI, and where there were two adoptive parents. Although the study included birth fathers, the sample size of birth fathers on the color evasive measure was insufficiently powered to include in the analyses. Dyads that included single adoptive parents were excluded to directly compare placement with couples. The study procedures were approved by the Institutional Review Board at the University of Oregon (IRB#04262013.034 and IRB#03042014.001). All birth and adoptive parent participants provided informed consent prior to participating in the research.

#### 2.1.1 Adoptive parents

Of the adoptive parent couples, there were 18 mother–mother couples, 11 father–father couples, and 354 mother–father couples. Adoptive parents were split into two groups: same-sex couples (*n* = 29) and mother–father couples (*n* = 354). Adoptive parents were predominately White people and were between 37.1 to 42.8 years old (SDs = 4.8–5.8) at the time of the child’s birth (see Table 1 for sample demographics).

**Table 1:** Sample demographics of mother–father and same-sex families.

	Mother–father families				Same-sex families			
	BM	BF	AM	AF	BM	BF	AM	AF
Age at TC birth (avg)	24.3 ± 6.1	25.8 ± 7.7	37.1 ± 5.4	38.1 ± 5.8	24.4 ± 5.9	30.1 ± 8.9	42.8 ± 5.3	38.6 ± 4.8
	(13–43)	(15–58)	(23–51)	(24–59)	(15–35)	(16–48)	(31–55)	(28–52)
Race/ethnicity	71.1% White people	70.7% White people	91.6% White people	89.4% White people	75.6% White people	76.5% White people	93.5% White people	97.2% White people
	13.3% Afr. Am.	11.2% Afr. Am.	3.5% Afr. Am.	5.1% Afr. Am.	12.2% Afr. Am.	17.6% Afr. Am.	4.3% Afr. Am.	2.8% Afr. Am.
	6.5% Hisp./Lat.	9.0% Hisp./Lat.	2.2% Hisp./Lat.	2.2% Hisp./Lat.	0.0% Hisp./Lat.	5.9% Hisp./Lat.	2.2% Hisp. Lat.	0.0% Hisp. Lat.
	4.4% Multi-eth	5.9% Multi-eth	1.0% Multi-eth	1.2% Multi-eth	7.3% Multi-eth	0.0% Multi-eth	0.0% Multi-eth	0.0% Multi-eth
	4.7% other^a^	3.2% other^a^	1.7% other^a^	2.1% other^a^	4.9% other^a^	0.0% other^a^	0.0% other^a^	0.0% other^a^
Median income at childbirth	20,000	20,000	110,000		14,550	20,000	110,000	137,500
Median income at CoBRAS	20,000	32,500	110,000		14,550	20,000	137,500	175,000
Median education attainment to date	High School degree	High School degree	4 year college or university	4 year college or university	High School degree	High School degree	Graduate program	Graduate program

BM, birth mother; BF, birth father; AM, adoptive mother; AF, adoptive father; TC, target child; CoBRAS, color-blind racial attitudes scale.

a American Indian/Alaskan Native, Asian, Native American/Other Pacific Islander, not reported.

#### 2.1.2 Birth mothers

The sample of birth mothers was more racially diverse than the adoptive families. Birth mother age at the time of the administration of the Color-Blind Racial Attitudes Scale ranged from 15 to 46 years (M = 27.86, SD = 6.27), and birth mother age at the time of the birth of the child ranged from 13 to 43 years (M = 24.09, SD = 6.08).

#### 2.1.3 Recruitment sites

Participants were recruited in collaboration with four recruitment sites in the Mid-Atlantic, West/Southwest, Midwest, and Pacific Northwest between March 2003 and January 2010. The recruitment sites worked with 45 adoption agencies, representing private, public, religious, and secular adoptions, in 15 states. None of the adoptions occurred through agencies that explicitly excluded same-sex couples from adopting.

### 2.2 Measures

#### 2.2.1 Color evasion

Birth mothers completed the Color-Blind Racial Attitudes Scale (CoBRAS) (Neville et al., 2000) using a web-based questionnaire or by phone interview between two to six years after the adoption placement. Neville et al. (2013) suggested CoBRAS remains stable across time, and thus it is reasonable to examine color evasion despite the gap between placement and completion of the CoBRAS. The CoBRAS captures “the belief that race should not and does not matter” (Neville et al., 2013, p. 60); as such, it reflects a worldview rather than specific actions taken by an individual. The total CoBRAS scale consists of 20 items and responses ranging from 1–6 (1 = Strongly Disagree, 6 = Strongly Agree; 2–5 are not labeled), with higher scores indicating more prominent color evasion (more racism). Example items include, “Everyone who works hard, no matter what race they are, has an equal chance to become rich,” and, “Racism may have been a problem in the past, but it is no longer an important problem.” The CoBRAS total scale (*α* = 0.60) indicated acceptable, albeit low, internal consistency in this sample. The original studies conducted to validate CoBRAS showed robust internal consistency for the CoBRAS total scale, with alphas ranging from .84 to .91 (Neville et al., 2000).

#### 2.2.2 Adoptive family type

Adoptive family type was measured via self-report by adoptive parents as a categorical variable with two categories: same-sex adoptive families (*n* = 29) and mother–father adoptive families (*n* = 354). We excluded single adoptive parents from the study to observe effects across two-parent households. Due to the small sample sizes of mother–mother and father–father family types, we collapsed mother–mother and father–father couples into one category: same-sex families. We coded family type as 0 = same-sex adoptive families and 1 = mother–father adoptive families.

#### 2.2.3 Covariates

##### 2.2.3.1 Birth mother race/ethnicity

Birth mother race/ethnicity was measured via self-report as a categorical variable: White people, African American/Black, Hispanic/Latina, American Indian/Alaskan Native, Asian, Native American/Other Pacific Islander, Multi-Ethnic, not reported. Due to small samples with each non-White people racial category, we coded birth mother race/ethnicity as 0 = non-White people (all other racial and ethnic identities; *n* = 105) and 1 = non-Latina White people (*n* = 278).

##### 2.2.3.2 Birth mother age

Birth mother age at the time of the administration of the CoBRAS was measured via self-report as a continuous variable (M = 27.86, SD = 6.27).

##### 2.2.3.3 Birth father level of involvement

The birth father’s level of involvement was measured via self-report by the birth mother answering the question, “How often did you talk with the birth father about the adoption process?” Responses were collected on a continuous scale of 1–5, where 1 = *Never*, 2 = *Rarely*, 3 = *Sometimes*, 4 = *Often*, and 5 = *Most of the Time* (M = 3.01, SD = 1.41).

### 2.3 Analytic approach

Analyses were conducted using SPSS version 28 (RRID: SCR_002865). A hierarchical logistic regression analysis was used to examine how CoBRAS predicted the choice of familial configuration (same-sex adoption placement versus mother–father adoption placement). We hypothesized that higher scores (i.e., attitudes characterized by stronger color evasion, reflecting more racial bias) would independently predict placement in mother–father parental configurations after accounting for all covariates. The hierarchical logistic regression model included birth mother race/ethnicity, birth mother age at the time of the administration of the CoBRAS, and level of birth father involvement in the adoption process as Step 1, and the CoBRAS total score as Step 2.

Birth mothers were also asked an open-ended question about how they chose the adoptive family and 12 birth mothers indicated that they did not choose a specific family. However, they still may have indicated priorities and preferences to the agency that were related to sexual identity. We ran a second analysis that excluded these 12 participants from the analysis, as a sensitivity analysis. All analyses, results, and tables were produced by the first author and verified for accuracy by the second author.

## 3 Results

### 3.1 Descriptive analyses

The means, standard deviations, and correlations among all study variables can be found in Table 2 and by family type in Table 3. The mean CoBRAS total score for birth mothers who placed their child with same-sex adoptive parents (M = 3.15, SD = 0.52) was lower than the mean CoBRAS total score for birth mothers who placed their child with mother–father adoptive parents (M = 3.38, SD = 0.54), which was in the expected direction of our hypothesis, *t*(381) = −2.18, *p* < 0.05. All correlations of major study variables were in the expected direction. The results of the sensitivity analysis that excluded birth mothers who indicated that they did not choose a specific adoptive family were similar to the full model (CoBRAS *β* = 0.627, Nagelkerke *R*^2^ = 0.05, *p* = 0.077).

**Table 2:** Correlations, means, and standard deviations for main study variables.

Variable	1	2	3	4	5
1. Birth mother age	–				
2. Birth mother race	−0.11*	–			
3. Birth father involvement	−0.04	0.13*	–		
4. CoBRAS	−0.002	0.02	−0.08	–	
5. Family type	0.06	−0.07	−0.06	0.11*	–
Mean	27.89	0.73	2.99	3.36	0.92
SD	6.24	0.45	1.40	0.54	0.26

CoBRAS, color-blind racial attitudes scale. CoBRAS ranged 1–6. BF involvement ranged 1–5. Race coded 0 = not White people, 1 = non-Latina White people. Family Type coded 0 = Same-Sex Family, 1 = Mother–Father Family. **p < 0.01 and *p < 0.05.

**Table 3:** Correlations and descriptive information for all study variables by family type.

Variable	1	2	3	4	M	SD
1. Birth mother age	–	−0.13*	−0.03	−0.03	27.99	6.28
2. Birth mother race	0.27	–	0.10*	0.03	0.72	0.45
3. Birth father involvement	−0.06	0.34	–	−0.06	2.96	1.39
4. CoBRAS	0.34	0.003	−0.24	–	3.37	0.54
Mean	26.59	0.83	3.28	3.15		
SD	5.53	0.38	1.49	0.52		

Correlations for same-sex families are below the diagonal and correlations for mother–father families are above the diagonal. CoBRAS, color-blind racial attitudes scale. CoBRAS ranged 1–6. BF involvement ranged 1–5. Race coded 0 = not White people, 1 = non-Latina White people. **p < 0.01 and *p < 0.05.

### 3.2 Birth mothers’ color evasion and adoptive family placement

Results of the logistic regression models indicated that the CoBRAS total score significantly predicted placement into the mother–father configurations (*β* = 0.74, *p* < 0.05), when controlling for birth mother race/ethnicity, birth mother age, and level of birth father involvement. Higher CoBRAS total scores decreased the likelihood of birth mothers placing children with same-sex couples by 2.03 times, supporting the study hypothesis. Nagelkerke *R*^2^ (an approximate measure of variability) indicated that the model may account for as much as 5% of the variance in family type placement in this model. See Table 4 for a summary of the results from the final regression model.

**Table 4:** Likelihood of placement with a mother–father couple by birth mothers’ CoBRAS total scores.

	Beta	Odds ratio	S.E.
Step 1			
Birth mother age	0.04	1.04	0.03
Birth mother ethnicity	−0.54	0.58	0.51
Birth father level of involvement	−0.14	0.87	0.14
Step 2			
Birth mother age	0.04	1.04	0.04
Birth mother ethnicity	−0.55	0.58	0.51
Birth father level of involvement	−0.11	0.90	0.14
CoBRAS total score	0.71*	2.03	0.34

Summary results from the final logistic regression model are shown here. Step 2 model −2 log likelihood = 197.34. *p < 0.05.

## 4 Discussion

Although there may be decreases in negative explicit attitudes towards sexual minorities across the United States (Westgate et al., 2015), negative implicit biases towards sexual minorities may still be widespread and contribute to discriminatory behaviors based on sexual orientation (Westgate et al., 2015). This study revealed that there was a negative association between color evasion—a proxy measure of implicit racial biases—and birth mothers’ adoption placement decisions. Specifically, birth mothers with higher color evasive attitudes were less likely to place their child with a same-sex couple during an open adoption process. The results indicate that bias in all its manifestations may be important to understand throughout the adoption process. The study does not suggest that the birth mothers involved in this study are actively or even consciously homophobic or racist in their attitudes. Rather, our study uncovers one way in which biases may interact with one another and manifest in systematic processes.

Birth mothers and the decisions they make about adoption placement are likely to be reflective of the norms of the particular society in which they live (Yngvesson, 2007; Sweeney, 2020). When making decisions regarding potential adoptive families for their children, birth mothers may be influenced by implicit and explicit biases that perpetuate social norms and contribute to the larger discourse of social norms. Similarly, birth mothers may choose adoptive parents who are “like them” and may have an unconscious bias toward placing their child(ren) with same-sex individuals or couples. Nonetheless, that about 8% of this study’s sample of adoptive families were same-sex couples is a sign that norms are changing quickly; in prior decades, agencies would not have allowed such placements.

Staats et al. (2017) remind us that adequate time is necessary for good decision-making, particularly when working through biases and attitudes that appear covert. Whenever possible, agencies should work with birth mothers to enable sufficient time and room for decisions to be made. Further, implicit associations may only be altered through the development of new associations (Dasgupta, 2013), and such alterations are inherently difficult to accomplish and even reversible (Greenwald et al., 2022). Thus, because of biases’ role in decision-making, agencies should consider the role biases play in their adoption processes, including the selection of which specific adoptive families are shared with a birth parent for consideration for the adoption placement. It is a topic for future discussions whether an agency or the adoption placement process is the appropriate place and time to actively work out homophobic and racial biases, but this is an important question to consider and not one to be taken lightly. Although agencies do not ban child placement with same-sex couples on a state level, biases by staff remain present, as evidenced by a study that found staff were apprehensive to place children with anyone that fell outside of the “heterosexual married family trope” (Pearson, 2017). Some racial and homophobic biases are, in fact, not implicit because they are overt or intentional, and this may become a source of tension and discomfort; at the same time, such biases may also get in the way of what may otherwise be sound adoption placement choices. For instance, endorsing color evasive bias (e.g., “I do not see race”) could intersect with other biases (e.g., “I do not see sexual orientation”), and these intersections could negatively impact all members of minoritized populations. This study focused exclusively on infant domestic adoptions, and it is necessary to examine whether similar patterns of association would be identified among children adopted at older ages, adopted from foster care, or in international adoptions.

### 4.1 Limitations and future research directions

Although the findings may indicate an interaction between racial attitudes and the decision to place infants with same-sex couples during the adoption process, several limitations should be noted. Despite using a large sample, there was a substantial difference in the sample sizes of the two family types (i.e., the dependent variable) and limited variability within some variables. Because regression uses a prediction-based model, a difference in sample sizes can make it difficult to detect significant associations. CoBRAS did not account for a great deal of variance in the current study; this may be attributable to the difference in sample sizes and should be explored in future studies. Given that women have been shown to be less biased than men toward gays and lesbians (Whitley and Kite, 1995), finding an association using a sample of all women was even more encouraging. Future studies may control for the influence of other beliefs or characteristics known to impact such bias (e.g., Averett et al., 2011). Future studies may also explore how variables such as birth mother ethnicity interplay with adoption decisions, an analysis that we did not have sufficient power to conduct. Similarly, due to limited variability in race/ethnicity data for adoptive parents in the current study, adoptive parent race/ethnicity was not included as a variable and should be a consideration in future research.

Because of the elapsed time between adoption and data collection of study measures (approximately 3 years), it is unclear whether color evasion influenced the placement decision or the placement decision and consequent experiences influenced color evasive attitudes. Although the CoBRAS stability tests show mixed results in terms of test–retest reliability (Neville et al., 2000), tests of the stability of several racial attitudes scales that separate measurement error from estimates of reliability have promising results (Cunningham et al., 2001). Additionally, although measures of internal consistency were moderate, they are consistent with results from the instrument’s initial validation (Neville et al., 2000), indicating that core racial ideologies may be relatively stable over time (Neville et al., 2013).

Another limitation of this study is that the adoption agencies used a variety of approaches for recruiting adoptive parents and for helping birth families throughout the selection process. Although our model controlled for birth mothers’ engagement in the selection process in a global way, the study remains limited by other circumstances (e.g., some agencies were located in regions of the United States where same-sex adoption is less common or discouraged). For example, although the states where the study took place permitted same-sex adoptions, residents in the specific city or town where a birth mother lived may have more prejudicial attitudes toward same-sex couples. Other unmeasured factors may include agency-level discrimination against same-sex couples or birth mothers not being offered the option of a same-sex couple due to other circumstances, such as fit. Future studies should aim to control for these circumstances, although doing so may also lead to skewed results in other ways. For example, excluding agencies that are located in regions that do not support same-sex adoption may create selection bias. Another limitation is that this study was conducted in the context of voluntary, domestic adoption processes. As a result, implications that extend beyond this type of adoption are speculative and require further study of phenomena in a variety of contexts.

Last, the measure used to capture color evasion is not a traditional measure of implicit attitudes. Measures such as the Implicit Association Task (Greenwald et al., 2009) may be better indicators of implicit biases, and future studies could use these implicit bias measures to further elaborate on this study’s findings. Another measurement limitation is that the analyses did not test the interaction between racial and sexual minority bias and how they affect adoption placement, as a measure of sexual minority bias was not available in the data set. A future study with a larger sample could examine how implicit racial bias moderates the relationship between implicit sexual minority bias and adoption placement to determine whether those with multiple forms of bias are more likely to engage in discriminatory behaviors.

## 5 Conclusion

Despite these limitations, to our knowledge, this is the first study to examine the role of color-evasion on decisions around selection of same-sex adoptive homes for one’s child. The results of this study indicate a number of considerations for theory and practice. One strength of this study is its inclusion of same-sex couples. Gates (2013) found that same-sex couples are proportionately more likely to raise adopted children than male–female couples. As such, the relevance of these findings is critical given the current and potential landscape of same-sex adoption. Given how common open adoptions are for domestic infant adoptions and, in recent years, other kinds of adoption (Grotevant, 2019), it is essential that we become increasingly aware of how our biases influence how we engage in decisions about child placement, particularly given the shortage of permanent adoptive homes for children adopted from foster care contexts. Furthermore, same-sex couples appear to be more likely to participate in open adoptions (Farr and Vázquez, 2020a).

Knowing that various forms of bias may intersect in subtle and complex ways, how can we use this knowledge to structure adoption processes in ways that minimize these interactions? Agencies could play a critical role in minimizing biases against same-sex couples through education efforts with their staff and acquiring knowledge of the extant literature on the general comparability of child and parent outcomes in straight and same-sex adoptive parent families. This study supports the preliminary suggestion that there may be a broader link between awareness/avoidance of racism and heterosexism—between structures of one bias and the manifestations of another. How does this implicate us at a community level, rather than at an individual one? In other words—and the case of adoption may just be one concrete example of many—how do we ensure that Crenshaw’s (1991) plea for attention to structures not be outranked by our desire for knowledge about the experience of bias? For example, bias reduction strategies (Staats et al., 2017) could be applied to the training of agency staff or social workers. Furthermore, approaches to the reduction of racism and other biases could be applied at the community level, which may have the potential to influence the decisions made by birth mothers during the adoption process. By understanding that these systems of inequity are intersectional and operate interdependently, we can work toward a complex understanding of bias and its manifestations, which may even provide us critical insight into how to dismantle it.

## Data availability statement

The data analyzed in this study is subject to the following licenses/restrictions: A de-identified dataset is available upon request to LL (leve@uoregon.edu). Requests to access these datasets should be directed to LL, leve@uoregon.edu.

## Ethics statement

The studies involving humans were approved by Institutional Review Board at the University of Oregon. The studies were conducted in accordance with the local legislation and institutional requirements. Written informed consent for participation in this study was provided by the participants’ legal guardians/next of kin.

## Author contributions

BR: Conceptualization, Formal analysis, Methodology, Writing – original draft, Writing – review & editing. DJ: Conceptualization, Methodology, Writing – review & editing, Visualization. AF: Conceptualization, Methodology, Writing – review & editing. DS: Investigation, Writing – review & editing, Project administration. JN: Investigation, Writing – review & editing, Funding acquisition, Project administration. JG: Writing – review & editing, Investigation, Project administration. LL: Writing – review & editing, Conceptualization, Formal analysis, Funding acquisition, Investigation, Methodology, Project administration, Supervision, Visualization.
